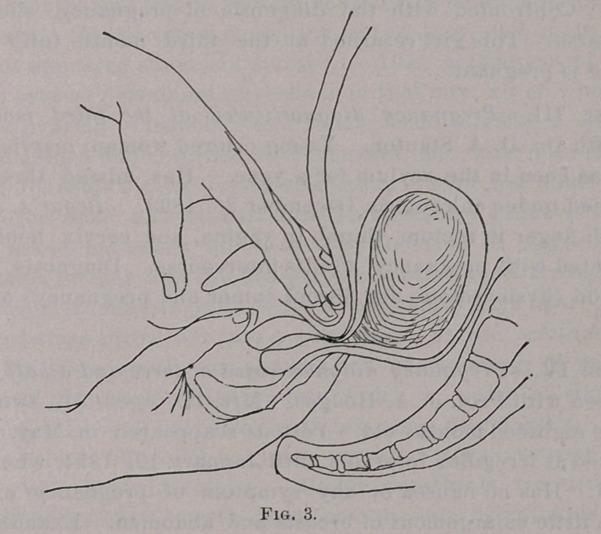# The Value of Hegar’s Sign of Pregnancy1Read at the annual meeting of the North Carolina State Medical Society, May 17, 1894.

**Published:** 1894-07

**Authors:** J. W. Long

**Affiliations:** Richmond, Va., Professor of Diseases of Women and Children in the Medical College of Virginia; 412 East Grace Street


Buffalo Medical and Surgical Journal

Vol. XXXIII. JULY, 1894. No. 12.

### Original Communications.

### THE VALUE OF HEGAR'S SIGN OF PREGNANCY.[1]

By J. W. LONG, M. D., Richmond, Va.,
Professor of Diseases of Women and Children in the Medical College of Virginia.

Perhaps the first problem which confronts every gynecologist, when called upon to diagnosticate the nature of a pelvic or abdominal tumor, is that of pregnancy. I am sure it is the first thing which enters my mind, for I have a painful recollection of being beguiled, by a designing woman, into passing a probe into a pregnant uterus, with the natural result of producing an abortion. This occurred when I was younger than I am now, when I knew more, and was more credulous of womankind.

Of course, to the man who is willing to simply wait "for Nature to take her course," it is immaterial as to whether a given tumor is a pregnant fundus or a fibroid, for "time will tell"; but to the man who feels the keen necessity of distinguishing the nature of these tumors, any sign which may be relied upon is a welcome addition to his resources.

The ordinary so-called certain signs of pregnancy develop so *late*, and many times not at all, that we turn with great expectation to a sign which promises to indicate with certainty the presence of pregnancy at an early period.

Hegar's sign consists essentially of *a softening and compressibility of the lower zone of the uterine body.* It has been erroneously stated to be a "softening of the upper part of the cervix"; but this is not true, for the hard cartilaginous-like cervix can be easily distinguished from the softened tissues above. The sign is obtained by bimanual palpation, the hands being placed in either of the several positions shown in the cuts taken from Sonntag, *American Journal of Obstetrics*, August, 1892.

1. Read at the annual meeting of the North Carolina State Medical Society, May 17, 1894.

Fig. 1 shows the intra-vaginal finger pressed into the anterior vaginal fornix, and the abdominal hand forced down behind the fundus and in front of the sacrum. In this way the *lower zone of the body of the uterus* is grasped between the opposing fingers, and it is astonishing to find how (apparently) thin the tissues are at this point. One can feel and distinguish the opposing fingers as readily as if only the lapel of his coat was interposed between them. I do not think one can fully realize how soft and bag-like the lower zone of the body of the pregnant uterus is until he has

FIG. 1.

actually seen, as well as touched, such a uterus while *in situ*. I had occasion to *see* and *feel* a three months pregnant uterus while doing a hysterectomy for fibrous tumors, complicated by pregnancy. This occurred in the practice of Drs. Cox and Staunton, of High Point, and is fully reported in the April number of the *Virginia Medical Monthly*.

So flaccid was the lower part of the uterus, that I would not believe it was not the half-full bladder, until a catheter was introduced, proving the bladder to be empty.

Fig. 2 illustrates the second method of obtaining this sign, the intra-vaginal finger being behind the cervix, and the abdominal hand being pushed down between symphysis and fundus.

Fig. 3 shows one hand on the abdomen between the symphysis and fundus, and one finger in the rectum, with the thumb in the vagina, controlling the cervix. This method may be facilitated by hooking down the cervix with a tenaculum. In fact, with the cervix

FIG. 2.

pulled down, the sign may be obtained by means of the thumb in the vagina and the finger in the rectum.

FIG. 3.

When the woman is very tender, or nervous, or the abdominal walls thick or rigid, it is necessary to employ anesthesia.

This paper is based upon the careful study of nine cases. The period when this sign may be observed, is from the eighth week to the sixth month. Other observers claim to have detected this sign at the fifth week. It hardly seems probable that the softening can be felt prior to this period, while after the beginning of the sixth month the uterine globe is so full that it cannot very well be compressed.

CASE I.—*Pregnancy diagnosticated at the eighth week.*—Case seen in connection with Prof. L. C. Bosher. Miss J., aged 19, having been led astray is anxious to know if she is pregnant. Says her periods have always been scanty and irregular. The last one appeared on March 10th and continued two days. Examination May 12th. *Hegar's sign* pronounced and made out by both Dr. Bosher and myself. The fundus is enlarged antero-posteriorly, which, of itself, is significant; breasts enlarged; areolar dark; papillæ elevated; patient complains of morning sickness and believes she is pregnant.

CASE II.—*Pregnancy diagnosticated at the ninth week.*—S. D., an intelligent colored girl of 18 years, single, no child, one abortion at third month, comes to get something to "bring on her spells." Says she has "took cold." Periods always regular, last one occurring November 24, 1893. Examination January 31, 1894. Slight cervical tear, os patulous and contains plug of mucus; mucosa soft. Lower zone of body compressible, the opposing fingers being easily felt through the uterus. Confronted with the diagnosis of pregnancy, she admitted intercourse. This girl returned at the third month fully convinced that she is pregnant.

CASE III.—*Pregnancy diagnosticated at the third month.*—Case seen with Dr. D. A. Stanton. Young colored woman; married, but husband has been in the asylum for a year. Has missed three periods. Examined under chloroform December 26, 1893. *Hegar's sign* made out with finger in rectum, thumb in vagina, and cervix hooked down. Confronted with pregnancy, admits intercourse. Diagnosis verified by operation (hysterectomy for fibrous tumor and pregnancy) on the same day.

CASE IV.—*Pregnancy diagnosticated at three and a half months.*—Case seen with Prof. J. A. Hodges. Mrs. H., aged 31, two children, last one eighteen months old. Periods reappeared in May, 1893, and continued at irregular intervals until January 10, 1894, when last one stopped. Has no nausea or any symptom of pregnancy, except, possibly, a little enlargement of breasts and abdomen. Examination May 2d. *Hegar's sign* pronounced. Vaginal discoloration, and cervical changes present. Fundus markedly enlarged.

CASE V.—*Pregnancy diagnosticated between the third and fourth months.*—Dispensary case. C. E., colored, aged 35 years, married eight years, four children, last pregnancy five years ago. Examined ——————. Has missed three periods. *Hegar's sign.* Small fibroid on posterior surface of fundus.

CASE VI. *Pregnancy diagnosticated between third and fourth months.* Dispensary case. J. J., colored, aged 27 years, single, three children, two abortions. Last pregnancy two years ago. Examined ——————. Has missed three periods. *Hegar's sign.*

This case was the subject of a clinical lecture delivered before the Summer school at the hospital of the Medical College of Virginia. Drs. J. Allison Hodges, George Ross, W. Augustus Lee and William P. Mathews were present, examined the patient, and all made out the sign without any trouble.

CASE VII.—*Pregnancy diagnosticated at the fourth month.*—This case was a private patient of Prof. Johnston's, and came with the evident purpose of deceiving him, as her history and sequel will show: Mrs. L. O., aged 25, married at 16, had a child one year later, aborted at third month one year after birth of child. About this time her husband left her and has not been with her since. Health good as a rule, except occasional attacks of kidney colic. Says that last June, while living in Philadelphia, she was accidentally pushed from a stoop and fell down six or eight stone steps, falling on her back, hurting her badly. Was in bed three months and had to be kept continually under opiates, so great was the pain. Her period came on the last of June, and again in July, but has not appeared since (six months). After getting out of bed she applied to several prominent physicians in that city, all of whom diagnosticated ovarian "trouble" or "tumor," and advised a celiotomy. This she declined and returned to Richmond, and later placed herself under Dr. Johnston's care, saying she had made up her mind to have the operation done, and desired him to operate. January 17th Dr. Johnston asked me to see the case with the view of diagnosis. The patient states further that she has paroxyms of pain occurring at irregular intervals, sometimes daily, sometimes several days apart; one just before Christmas threw her into a convulsion, as did another one on January 1st, since which time she has had no paroxysm of pain. She complains of profuse fetid leucorrhea, which is worse at times. She was so tender I could not make a satisfactory examination. I could discover a tumor, but could not make out what it was, so with her consent we gave her chloroform. Under anesthesia we determined: absence of leucorrhea, cervix hard, mucosa soft, os patulous and contains plug of mucus, fundus size of four months pregnancy, *Hegar's sign marked*, fibroid size of lemon and with broad face situated on right side of fundus. This patient was of such good standing that it was with difficulty I persuaded Dr. Johnston to confront her with pregnancy, but finally he did. She denied it indignantly, but the doctor was emphatic, and finally she admitted intercourse in September, just four months prior to date of examination. She was sent out of the hospital and miscarried ten days afterward.

CASE VIII.—*Pregnancy diagnosticated at four and a half months.*—This case also occurred in Dr. Johnston's service. Mrs. H., age about 30, a most excellent lady, mother of one child two years old. The only periods she has had since the birth of her child occurred in April, July and October (15th) of last year. For nearly a year she has suffered with pain and soreness in left ovarian region, now has a marked abdominal enlargement. She has had no morning sickness, no enlargement of breasts, no appearance of milk, indeed nothing to cause her to suspect pregnancy. March 6th, Dr. Johnston asked me to examine her. It was necessary to use chloroform. We found: cervix hard, mucosa soft, os patulous and contains plug of mucus, fundus enlarged, *Hegar's sign* present, left cystic oöphoro-salpingitis. Both patient and husband were delighted with the diagnosis of pregnancy, which was confirmed two weeks later by quickening.

CASE IX.—*Pregnancy of five or six months in which I failed to get the sign.*—Case seen with Dr. Bulla, of Randolph. Mrs. K., a lady living in North Carolina; age 35; married sixteen months; no children; periods always regular and very profuse until June, 1893, the flow was scanty, but began again in July and was nearly continuous until the middle of October, when she passed a large quantity of clots and shreds, whereupon her abdomen, which was the size of a four or five months pregnancy, rapidly subsided to normal size. After this her periods did not appear again until February, since when she has had a continuous bloody flow. She has had no enlargement of breasts, no nausea, and does not believe she is pregnant. Examination, March 17th, under chloroform, when we discovered: cervix pushed to left side, mucosa soft, os patulous, fundus size of four and a half or five months pregnancy, rythmical contractions of fundus, hard tumor size of fundus, situated behind and to the right, numerous small tumors in and around upper part of cervix and lower zone of body, Hegar's sign could not be elicited, due either to presence of the tumors, or to the advance stage of pregnancy. Diagnosis: pregnancy and fibroid tumors. The second night afterward the woman miscarried.

My conclusions then are:

1. That between the second and fifth months Hegar's sign of pregnancy is one of great value, its presence always indicating pregnancy.

2. It is applicable to any case where the abdominal walls are thin and flaccid enough to grasp the uterus between the two hands, as detailed above.

3. Fibroid tumors are the most misleading complication (two cases, *supra*).

4. Anesthesia is often necessary.

Since reading this paper before the North Carolina Medical Society, at Greensboro, May 17, 1894, I was asked to examine:

CASE X. Mrs. H.; age, 22; periods painful and profuse; last one, February 20th, lasting four or five days. Never pregnant and does not believe she is pregnant now. Examination under chloroform, May 18th, found *Hegar's sign*; also mucosa soft, fundus size of three months pregnancy and areola slightly darker than normal. Diagnosis: *pregnancy of three months.*

412 EAST GRACE STREET.

## Figures and Tables

**Fig. 1. f1:**
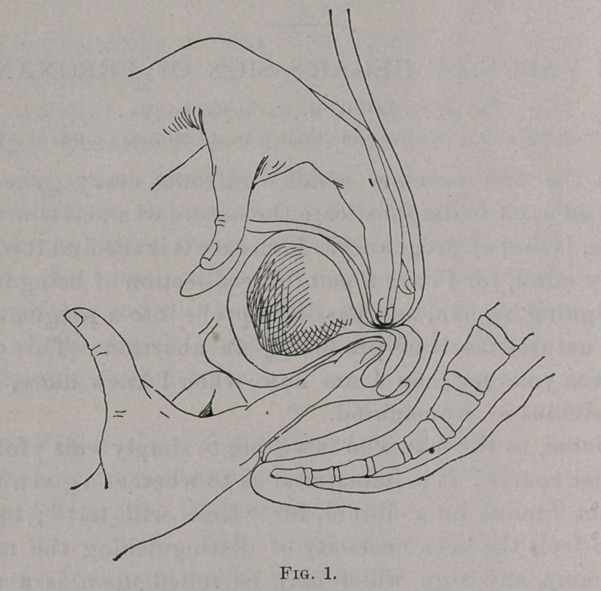


**Fig. 2. f2:**
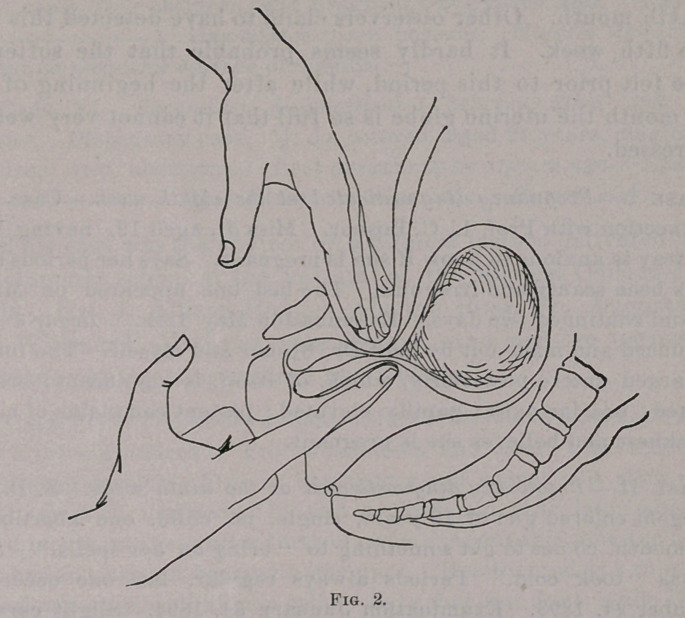


**Fig. 3. f3:**